# Nicotinamide mononucleotide supplementation reverses vascular dysfunction and oxidative stress with aging in mice

**DOI:** 10.1111/acel.12461

**Published:** 2016-03-11

**Authors:** Natalie E. de Picciotto, Lindsey B. Gano, Lawrence C. Johnson, Christopher R. Martens, Amy L. Sindler, Kathryn F. Mills, Shin‐ichiro Imai, Douglas R. Seals

**Affiliations:** ^1^ Department of Integrative PhysiologyUniversity of Colorado BoulderBoulderCO; ^2^ Department of Developmental BiologyWashington University School of MedicineSt. LouisMOUSA; ^3^ Present address: Department of Pharmaceutical SciencesSchool of PharmacyUniversity of ColoradoDenverCOUSA; ^4^ Present address: Department of Health and Human PhysiologyUniversity of IowaIowa CityIAUSA

**Keywords:** arterial stiffness, endothelial dysfunction, NAD+, oxidative stress, SIRT1

## Abstract

We tested the hypothesis that supplementation of nicotinamide mononucleotide (NMN), a key NAD ^+^ intermediate, increases arterial SIRT1 activity and reverses age‐associated arterial dysfunction and oxidative stress. Old control mice (OC) had impaired carotid artery endothelium‐dependent dilation (EDD) (60 ± 5% vs. 84 ± 2%), a measure of endothelial function, and nitric oxide (NO)‐mediated EDD (37 ± 4% vs. 66 ± 6%), compared with young mice (YC). This age‐associated impairment in EDD was restored in OC by the superoxide ($O_{2}^{-}$) scavenger TEMPOL (82 ± 7%). OC also had increased aortic pulse wave velocity (aPWV, 464 ± 31 cm s^−1^ vs. 337 ± 3 cm s^−1^) and elastic modulus (EM, 6407 ± 876 kPa vs. 3119 ± 471 kPa), measures of large elastic artery stiffness, compared with YC. OC had greater aortic $O_{2}^{-}$ production (2.0 ± 0.1 vs. 1.0 ± 0.1 AU), nitrotyrosine abundance (a marker of oxidative stress), and collagen‐I, and reduced elastin and vascular SIRT1 activity, measured by the acetylation status of the p65 subunit of NFκB, compared with YC. Supplementation with NMN in old mice restored EDD (86 ± 2%) and NO‐mediated EDD (61 ± 5%), reduced aPWV (359 ± 14 cm s^−1^) and EM (3694 ± 315 kPa), normalized $O_{2}^{-}$ production (0.9 ± 0.1 AU), decreased nitrotyrosine, reversed collagen‐I, increased elastin, and restored vascular SIRT1 activity. Acute NMN incubation in isolated aortas increased NAD ^+^ threefold and manganese superoxide dismutase (MnSOD) by 50%. NMN supplementation may represent a novel therapy to restore SIRT1 activity and reverse age‐related arterial dysfunction by decreasing oxidative stress.

## Introduction

Cardiovascular diseases (CVD) remain the leading cause of morbidity and mortality in developed nations (Mortality & Causes of Death, 2014), and advancing age is the primary risk factor for CVD (Lakatta & Levy, 2003). The number of older adults in the developed world is expected to at least double by 2050 (Petsko, 2008), and this is associated with projections of marked increases in CVD burden (Heidenreich *et al*., 2011). Therefore, there is an urgent need to develop treatments that reduce the risk of CVD with aging.

Two key antecedents and independent predictors of clinical CVD in older adults are vascular endothelial dysfunction, assessed by endothelium‐dependent dilation (EDD), and large elastic artery stiffness, measured by aortic pulse wave velocity (aPWV) (Lakatta & Levy, 2003; Deanfield *et al*., 2007; Yeboah *et al*., 2007; Mitchell *et al*., 2010). A common mechanism that contributes to both vascular endothelial dysfunction and large elastic artery stiffness with aging is excessive superoxide‐associated vascular oxidative stress (Seals *et al*., 2011; Fleenor *et al*., 2012a; Bachschmid *et al*., 2013). Increased vascular production of superoxide occurs with aging and reduces the bioavailability of the vasoprotective and vasodilatory molecule nitric oxide (NO), while also causing alterations in major structural proteins (collagen and elastin) in the large elastic arteries (i.e., the aorta and carotid arteries) (Deanfield *et al*., 2007; Fleenor *et al*., 2010; Seals *et al*., 2014). These changes contribute directly to age‐related endothelial dysfunction and increased arterial stiffness (Lakatta & Levy, 2003; Seals *et al*., 2011). As such, treatments that reduce the excessive superoxide production in aging arteries hold the potential for improving age‐associated vascular dysfunction.

We have previously shown that lifelong caloric restriction (CR), as well as short‐term CR in old animals, prevents or reverses endothelial dysfunction and large elastic artery stiffening by reducing superoxide production, increasing NO bioavailability, and modifying structural proteins (Rippe *et al*., 2010; Donato *et al*., 2013). However, because adherence to CR is not practical for most humans, there is growing interest in pharmacological therapies that may induce the benefits of CR. In this context, the mammalian SIRT1, one of seven members in the sirtuin family of protein deacetylases/deacylases, is a nicotinamide adenine dinucleotide (NAD^+^)‐dependent deacetylase that acts as a metabolic energy sensor implicated in several of the beneficial effects of CR, including reduced oxidative stress (Boily *et al*., 2008; Merksamer *et al*., 2013). Our previous work shows that reduced SIRT1 expression and activity is a key mechanism mediating impaired EDD in aging arteries (Rippe *et al*., 2010; Donato *et al*., 2011; Gano *et al*., 2014), and our recent findings indicate that pharmacological activation of SIRT1 with the compound SRT1720 improves EDD in old mice in part by reducing oxidative stress (Gano *et al*., 2014).

Supplementation with nicotinamide mononucleotide (NMN), a key NAD^+^ intermediate, activates SIRT1 and improves metabolic and stress responses with aging in mice (Yoshino *et al*., 2011; Gomes *et al*., 2013). Thus, NMN is viewed as a promising therapy for age‐associated physiological dysfunction and disease (Imai, 2010; Yoshino *et al*., 2011). However, it is unknown whether NMN supplementation can increase arterial SIRT1 activity, reduce vascular oxidative stress, and reverse vascular dysfunction with aging. In the present study, we tested this hypothesis, while also assessing the effects of NMN on vascular superoxide production, NO bioavailability, and collagen and elastin expression. Acetylation status of the p65 subunit of nuclear factor kappa B (NFκB), a well‐established target of SIRT1, was used to determine activation of the enzyme, as described previously (Yoshino *et al*., 2011; Gano *et al*., 2014).

## Results

### Animal characteristics and NMN intake

Selected morphological characteristics are shown in Table 1. There were no differences in body mass across the four groups. We observed an age‐related increase in heart mass and a decrease in fat and muscle mass, which were not altered with NMN treatment. All animals consumed the same quantity of food throughout the duration of the study, and average NMN intake was similar in both young and old treated groups (data not shown).

**Table 1:** Animal Characteristics

	YC	OC	YNMN	ONMN
Body mass (g)	30.1 ± 0.7	30.3 ± 0.6	28.0 ± 0.9	30.8 ± 0.6
Heart mass (mg)	145 ± 6	176 ± 7*	139 ± 7	182 ± 8*
Quad mass (mg)	176 ± 5	151 ± 4*	185 ± 6	146 ± 6*
Gastroc mass (mg)	151 ± 3	124 ± 3*	148 ± 8	126 ± 2*
WAT mass (mg)	730 ± 65	472 ± 41*	600 ± 35	430 ± 50*
SubQ fat mass (mg)	334 ± 32	207 ± 17*	252 ± 16	201 ± 23*

Values are mean ± SEM *P < 0.05 vs. YC. *n* = 13–19/group.

Quad, quadriceps; Gastroc, gastrocnemius; WAT, white adipose tissue; SubQ, subcutaneous fat.

### NMN treatment restores maximum EDD and NO‐mediated EDD in old mice

**Figure 1:**
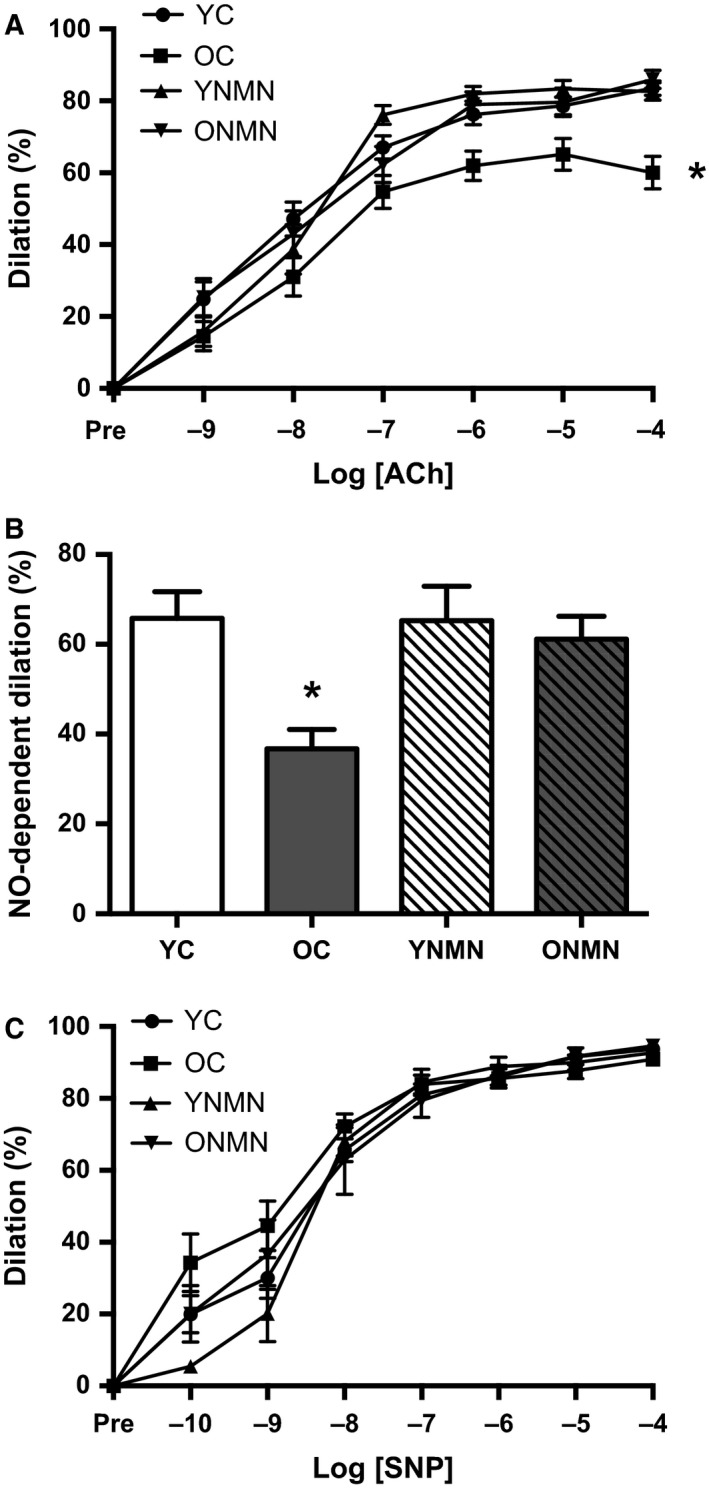
Endothelium‐dependent, nitric oxide (NO)‐dependent, and endothelium‐independent dilation. (A) Dose–responses to the endothelium‐dependent dilator acetylcholine (ACh) in young and old control (YC and OC) and young and old NMN‐supplemented (YNMN and ONMN) mice (*n* = 13–22 per group). (B) NO‐dependent dilation (Max DilationACh − Max DilationACh + l‐NAME) (*n* = 5–12 per group). (C) Dose–responses to the endothelium‐independent dilator sodium nitroprusside (SNP) (*n* = 7–22 per group). Values are mean ± SEM. *P <* *0.05 vs. all.

Baseline carotid artery diameters (μm) assessed *ex vivo* were greater in old mice (controls: 480 ± 17; treated: 478 ± 9) vs. young mice (controls: 424 ± 4; treated: 432 ± 5), as we have previously reported (Sindler *et al*., 2011). NMN supplementation had no effect on baseline carotid artery diameters. Maximum EDD to acetylcholine assessed *ex vivo* was lower in old control compared with young control mice and was mediated in part by a diminished NO dilatory influence, as indicated by a smaller reduction in EDD in the presence vs. absence of the NO synthase inhibitor N‐G‐nitro‐L‐arginine‐methyl ester (l‐NAME) (Fig. 1A,B). NMN supplementation rescued EDD in old mice by restoring NO‐mediated dilation, but had no effect in young treated animals (Fig. 1A,B). There were no differences in maximal EDD or NO‐mediated EDD in young and old treated animals vs. young controls (Fig. 1 A,B). Endothelium‐independent dilation to the NO donor sodium nitroprusside, a control to assess vascular smooth muscle sensitivity to NO, was not different among the groups (Fig. 1C).

### NMN reduces vascular oxidative stress

**Figure 2:**
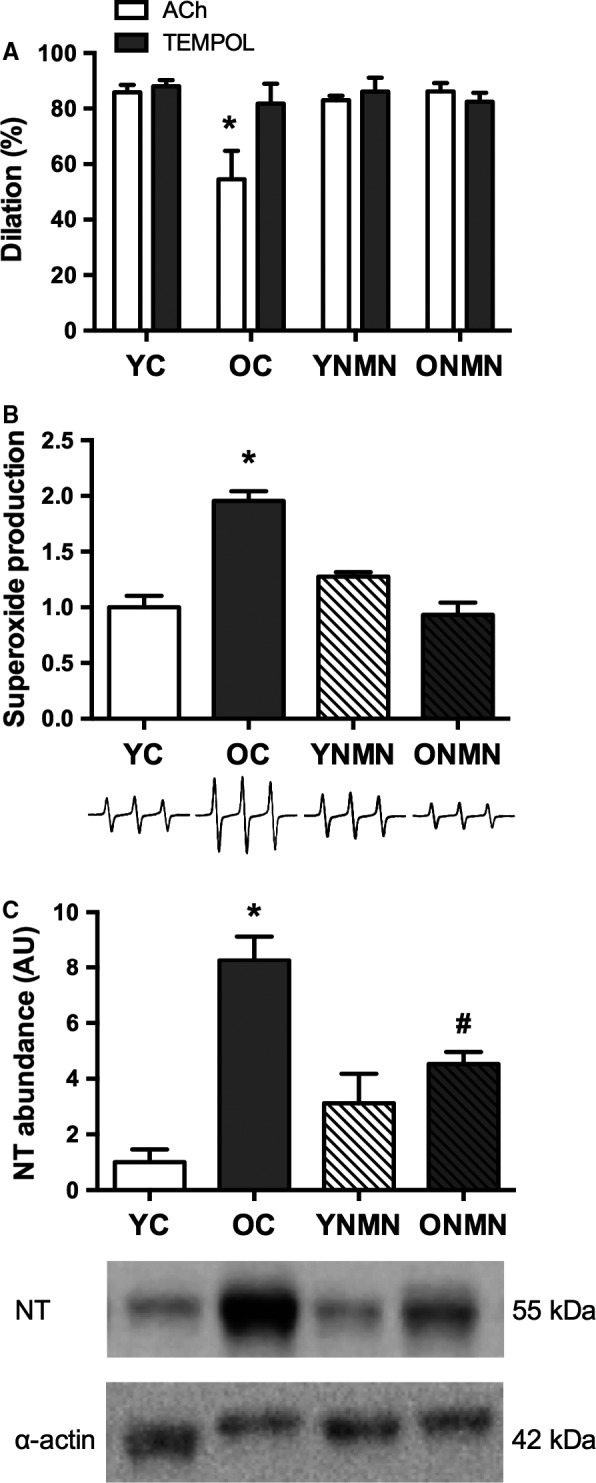
Vascular oxidative stress. (A) Maximal dose–response to the endothelium‐dependent dilator acetylcholine (ACh) in young and old control (YC and OC) and young and old NMN‐supplemented (YNMN and ONMN) mice in the presence or absence of TEMPOL (*n* = 5–9 per group) *P < 0.05 vs. TEMPOL. (B) Superoxide production, assessed by electron paramagnetic resonance (EPR). Values are normalized to YC mean value. Representative EPR signal below (*n* = 4–9 per group). (C) Nitrotyrosine (NT) abundance in aorta. Data are expressed relative to α‐smooth muscle actin and normalized to YC mean value. Representative Western blot images below (*n* = 4–6 per group). Values are mean ± SEM. *P <* *0.05 vs. all; # *P *<* *0.05 vs. YC.

*Ex vivo* incubation with the superoxide dismutase mimetic, 4‐Hydroxy‐2,2,6,6‐tetramethylpiperidine‐1‐oxyl (TEMPOL), restored EDD in carotid arteries of old control animals, while having no effect in the other groups (Fig. 2A), indicating excessive superoxide‐mediated endothelial dysfunction with aging. To further assess the influence of aging and NMN treatment on oxidative stress in arteries, aortas were used because of the greater amount of tissue provided for biochemical assay, as described previously (Sindler *et al*., 2011; Donato *et al*., 2013; LaRocca *et al*., 2013; Gano *et al*., 2014). Consistent with the results of the pharmacological‐function experiments with TEMPOL, compared with young mice, aortas from old animals exhibited increased superoxide production, directly assessed by electron paramagnetic resonance (EPR) spectroscopy (Fig. 2B), and increased nitrotyrosine abundance (Fig. 2C), a marker of oxidative protein modification and cellular footprint of oxidative stress. NMN treatment ameliorated the age‐related increase in superoxide production (Fig. 2B) and markedly reduced aortic nitrotyrosine abundance in old mice (Fig. 2C), while having no significant effect in young mice. Together, these data indicate that NMN reverses age‐associated oxidative stress in arteries, which, in turn, mediates improvements in endothelial function.

### NMN treatment normalizes aortic stiffness in old mice

**Figure 3:**
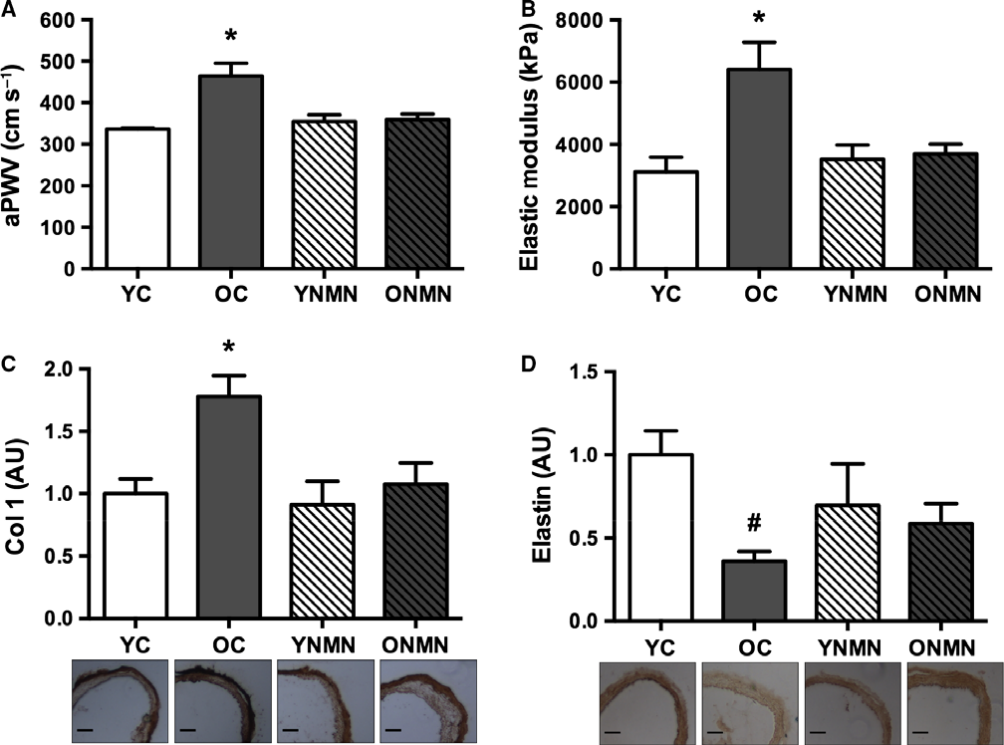
Large elastic artery stiffness. (A) Aortic pulse wave velocity (aPWV) in young and old control (YC and OC) and young and old NMN‐supplemented (YNMN and ONMN) mice (*n* = 5–9 per group). (B) Elastic modulus (*n* = 5–6 per group). (C) Total collagen 1 (Col 1) expression in aorta (*n* = 4–9 per group). (D) Total elastin expression in aorta. Values normalized to YC mean value. Representative images below (*n* = 4–11 per group). Values are mean ± SEM. Bars = 100 μm. *P <* *0.05 vs. all; # *P *<* *0.05 vs. YC.

Large elastic artery stiffness, as assessed *in vivo* by aPWV, was greater in old control compared with young control mice (Fig. 3A). NMN treatment reversed the age‐associated increase in aPWV in old mice, while having no effect in young mice (Fig. 3A). Similarly, the elastic modulus, an *in vitro* index of intrinsic arterial stiffness, was higher in old controls compared with young and was normalized with NMN supplementation (Fig. 3B). Thoracic aortas from old control animals exhibited markedly increased collagen type I expression (Fig. 3C) and diminished elastin (Fig. 3D) compared with young controls. In old mice, NMN reduced arterial collagen type I to levels of young mice (Fig. 3C), and increased elastin to levels not significantly different from young mice (Fig. 3D). Together, these observations indicate that NMN reverses large elastic artery stiffening with aging, in part by normalizing collagen and partially preserving elastin in the arterial wall.

### NMN activates arterial SIRT1

**Figure 4:**
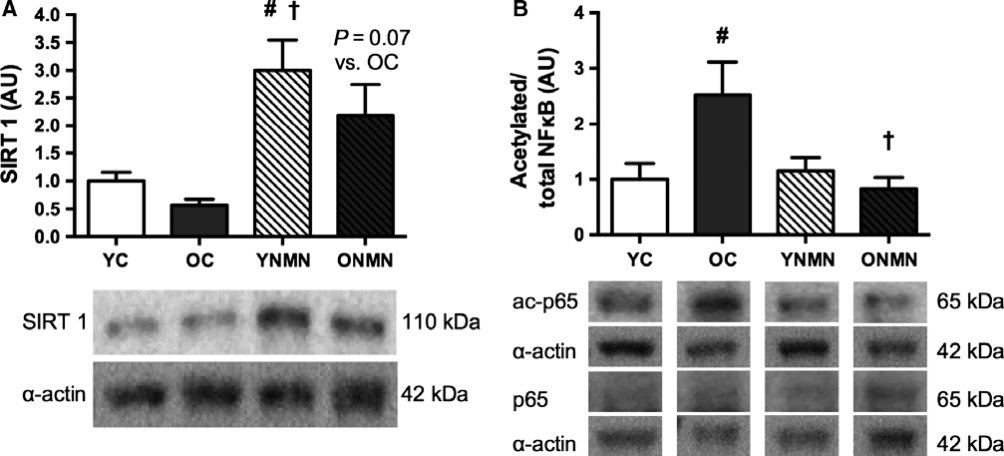
SIRT1 expression and activity. (A) SIRT1 expression in aorta of young and old control (YC and OC) and young and old NMN‐supplemented (YNMN and ONMN) mice (*n* = 5–7 per group). (B) Ratio of acetylated to total NFκB in aorta (*n* = 5–11 per group). Data are expressed relative to α‐smooth muscle actin and normalized to YC mean value. Representative Western blot images below. Values are mean ± SEM. #*P *<* *0.05 vs. YC; †*P *<* *0.05 vs. OC.

Mean levels of aortic SIRT1 expression were ~50% lower in the old animals, compared with young mice, although the difference did not reach statistical significance (Fig. 4A). NMN supplementation increased SIRT1 protein expression in young animals and tended to increase SIRT1 in old animals (Fig. 4A). The p65 subunit of NFκB is a major target of SIRT1 and is deacetylated in response to SIRT1 activation (Yeung *et al*., 2004). As such, SIRT1 activation was determined by assessing the ratio of acetylated to total NFκB (p65 subunit). This ratio was markedly higher in aorta of old control animals compared with young controls, indicating that aortic SIRT1 activity was reduced with aging. Most importantly, NMN supplementation restored aortic SIRT1 activity in old animals (Fig. 4B).

**Figure 5:**
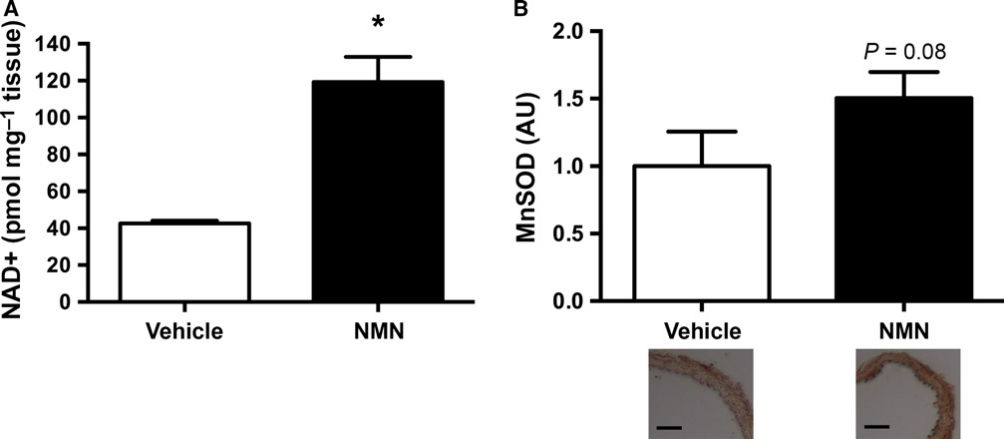
NAD^+^ and MnSOD. (A) NAD^+^ production in aortas from young mice incubated *in vitro* with vehicle or NMN for 48 h (*n* = 3–5 per group). (B) Manganese superoxide dismutase (MnSOD) expression in aortas from old mice incubated *in vitro* with vehicle or NMN for 48 h (*n* = 4–5 per group). Values normalized to vehicle mean value. Representative images below. Values are mean ± SEM. Bars = 100 μm. *P <* *0.05.

We were unable to detect an increase in aortic NAD^+^ concentration in animals chronically supplemented with NMN. This was possibly because the mice were consuming small amounts of NMN in the drinking water throughout the day, and although this NMN was taken up by the vascular tissue, we posit that the small quantities of NAD^+^ produced were rapidly metabolized. To determine whether NMN is capable of increasing arterial NAD^+^ bioavailability, we incubated isolated aortic tissue, *in vitro*. Incubation of aortic segments from young mice with NMN (100 μm) for 48 h resulted in a threefold higher production of NAD^+^ compared with vessels incubated in Dulbecco's modified Eagle medium (DMEM) control media (Fig. 5A).

We also took advantage of these *in vitro* experiments to gain insight into a likely mechanism by which NMN may have reduced vascular oxidative stress *in vivo*. To do so, we assessed the mitochondrial antioxidant, manganese superoxide dismutase (MnSOD), which is upregulated in response to SIRT1 activation, possibly through stimulation of peroxisome proliferator‐activated receptor‐gamma coactivator‐1alpha (PGC1α) (Pfluger *et al*., 2008). We found that aortas from old mice incubated in NMN for 48 h had a 50% higher staining of MnSOD compared with aortas incubated in control media (Fig. 5B).

Collectively, these data suggest that NMN is capable of increasing NAD^+^ bioavailability in the vasculature and this likely explains the observed increase in arterial SIRT1 activity. Because MnSOD is upregulated in arteries incubated in NMN, this may also represent a mechanism by which oxidative stress was reduced in old animals supplemented with NMN in the drinking water.

## Discussion

In the present study, 8 weeks of NMN supplementation restored a marker of arterial SIRT1 activity and ameliorated age‐associated endothelial dysfunction and large elastic artery stiffening in male C57Bl/6 mice. These improvements were associated with restored NO bioavailability, reduced oxidative stress, and complete or partial normalization of structural proteins in the arterial wall. Overall, our findings provide the first evidence supporting the promising translational potential of NMN supplementation for the treatment of arterial aging.

### NMN improves NO‐mediated EDD and reduces arterial oxidative stress

Endothelial dysfunction is the major antecedent to atherosclerosis, a predictor of clinical CVD risk, and is linked to many common disorders of aging including cognitive impairments, Alzheimer's disease, motor dysfunction, insulin resistance, and sarcopenia (Heitzer *et al*., 2001; Gokce *et al*., 2002; Lakatta & Levy, 2003; Widlansky *et al*., 2003; Seals *et al*., 2011, 2014). We have previously demonstrated that arterial SIRT1 activity is reduced with aging and contributes to the age‐related impairment in endothelial function (Donato *et al*., 2011; Gano *et al*., 2014). We and others also have shown that lifelong CR prevents (Csiszar *et al*., 2009; Donato *et al*., 2013) and short‐term CR reverses (Rippe *et al*., 2010) the age‐associated decline in endothelial function and that these effects are associated with enhanced arterial SIRT1 activity. Consistent with these observations, we recently found that 4 weeks of treatment with the SIRT1 activator SRT1720 restores endothelial function (i.e., EDD) in old mice (Gano *et al*., 2014).

In the present study, we demonstrate a reversal of age‐associated endothelial dysfunction by oral NMN supplementation. As established in our previous work (Rippe *et al*., 2010; Sindler *et al*., 2011; Fleenor *et al*., 2013; LaRocca *et al*., 2013), aging in the control animals was associated with an impairment in *ex vivo* carotid artery EDD in response to acetylcholine due to reduced NO‐mediated dilation (Fig. 1A,B). The latter was determined as the difference in EDD in the absence vs. presence of NO production achieved by co‐administration of the NO synthase inhibitor, l‐NAME. NMN supplementation completely restored EDD by restoring NO‐mediated dilation. The improvement in EDD with NMN treatment in old mice was not due to an increase in vascular smooth muscle sensitivity to NO because NMN did not influence dilation in response to administration of a NO donor (sodium nitroprusside), that is, a dilation that is not induced by endothelial NO production (Seals *et al*., 2011).

Our results also suggest that the effects of aging and NMN supplementation on endothelial function were mediated by differences in oxidative stress. Consistent with our previous work (Rippe *et al*., 2010; Sindler *et al*., 2011; Fleenor *et al*., 2013; Gano *et al*., 2014), pharmacofunctional experiments revealed that arteries from old untreated mice showed a complete restoration of maximal EDD in the presence of the superoxide dismutase mimetic, TEMPOL, indicating that excessive superoxide‐related oxidative stress was the cause of the age‐associated impairment of EDD (Fig. 2A). Importantly, in contrast to the untreated old mice, TEMPOL caused no improvement in EDD in old mice receiving NMN treatment, suggesting that NMN supplementation restored EDD in the old animals by abolishing superoxide‐mediated suppression of endothelial function with aging. This finding is in agreement with other interventions that restore EDD with aging (Rippe *et al*., 2010; Sindler *et al*., 2011; Fleenor *et al*., 2013; Gano *et al*., 2014).

These function‐based observations are supported by our direct assessments of arterial superoxide production using EPR spectroscopy, as well as our results for nitrotyrosine, a well‐established cellular marker of oxidative stress (Csiszar *et al*., 2002; Sindler *et al*., 2011; Fleenor *et al*., 2012a; Donato *et al*., 2013). Aging in control animals was associated with marked increases in both aortic superoxide production and nitrotyrosine abundance (Fig. 2B,C), as we have reported previously (Rippe *et al*., 2010; Sindler *et al*., 2011; Fleenor *et al*., 2012a, 2013; Donato *et al*., 2013; LaRocca *et al*., 2013). In response to NMN supplementation, we observed a normalization of aortic superoxide production and a marked reduction in nitrotyrosine abundance in old mice. Moreover, arteries incubated in NMN for 48 h had a 50% increase in MnSOD staining (Fig. 5B), suggesting that the reduction in oxidative stress is likely due, in part, to an increase in antioxidant defense mechanisms.

Collectively, these findings support the hypothesis that oxidative stress is a key contributor to age‐associated endothelial dysfunction and suggest that the suppression of oxidative stress may be a major mechanism by which NMN exerts its beneficial effects on endothelial function in old animals. These observations are consistent with our previous findings in young and old mice subjected to a similar duration of CR (Rippe *et al*., 2010). Moreover, as recently reported by our laboratory, the SIRT1 activator, SRT1720, may also be effective in reversing age‐associated endothelial dysfunction by reducing oxidative stress, albeit with different effects on NO (Gano *et al*., 2014).

### NMN reduces large elastic artery stiffness

Increased stiffening of the large elastic arteries occurs with advancing age and is a major independent risk factor for age‐associated CV events and clinical CVD (Reddy *et al*., 2003; Sutton‐Tyrrell *et al*., 2005; Fleenor *et al*., 2012b). Increased aortic stiffness, in particular, reduces the ability to buffer increases in pressure produced by systolic ejection of blood into the large elastic arteries with each cardiac contraction. This increases systolic blood pressure and arterial pulse pressure (the difference between systolic and diastolic blood pressure), as well as the ‘pulsatility’ of blood flow, which is transmitted to the microvasculature of vulnerable high‐flow organs such as the brain and kidney, causing end‐organ damage and other pathophysiological effects (Lakatta & Levy, 2003; Mitchell *et al*., 2010; Mitchell, 2014). Here, we show that 8 weeks of NMN supplementation reverses the age‐associated increase in two functional indices of aortic stiffness: aPWV, the gold standard clinical measure of large elastic artery stiffness (Mitchell *et al*., 2010; Vlachopoulos *et al*., 2010), and the elastic modulus, an *in vitro* measure of the intrinsic mechanical properties of arteries (Humphrey, 2002; Akhtar *et al*., 2011; Fleenor *et al*., 2012b) (Fig. 3A,B).

The mechanisms by which arteries stiffen with age are not completely understood, but are thought to include changes in the composition of structural proteins within the arterial wall. Collagen (type I) is the primary load‐bearing protein in the arterial wall, and its abundance is increased with advancing age (Zieman *et al*., 2005; Diez, 2007; Fleenor *et al*., 2012b). In contrast, elastin, the main structural protein conferring elasticity, is reduced in old arteries (Zieman *et al*., 2005; Diez, 2007; Fleenor *et al*., 2012b). We have previously shown in mice (Fleenor *et al*., 2012a) and cultured aortic fibroblasts (Fleenor *et al*., 2010) that oxidative stress contributes to some or all of the age‐associated structural changes seen within the arteries. In the present study, we demonstrate that 8 weeks of NMN supplementation reverses the accumulation of whole‐vessel collagen type I and enhances arterial elastin in old mice (Fig. 3C,D), suggesting that NMN reduces arterial stiffness, at least in part, by ameliorating these structural changes that occur to arteries with advancing age.

We have observed reductions in aortic collagen in old mice subjected to other short‐term late‐life behavioral or pharmacological interventions (Fleenor *et al*., 2010, 2012a, 2013; LaRocca *et al*., 2013), although not in every case (Fleenor *et al*., 2012b). Interestingly, NMN also induced a partial restoration of aortic elastin to levels not significantly different from young control animals. We have not previously observed an increase in aortic elastin with any other late‐life lifestyle or pharmacological intervention in mice (Fleenor *et al*., 2010, 2012a,b), although lifelong CR protects against the loss of elastic properties within the arteries, including elastin degradation (Fornieri *et al*., 1999; Donato *et al*., 2013). Thus, it is possible that NMN may partially restore the loss of arterial elastin by mimicking the effects of CR, although further studies would be needed to provide direct support for this mechanism.

### Activation of SIRT1 by NMN

NAD^+^ bioavailability decreases with age in a number of mammalian tissues (Yoshino *et al*., 2011; Gomes *et al*., 2013; Stein & Imai, 2014), and administration of NMN increases NAD^+^ bioavailability in preclinical models of aging (Yoshino *et al*., 2011; Gomes *et al*., 2013; Stein & Imai, 2014). Enhancing NAD^+^ biosynthesis with NAD^+^ precursors such as NMN and nicotinamide riboside increases the activity of the NAD^+^‐dependent deacetylase SIRT1 (Imai, 2010; Satoh *et al*., 2011; Yoshino *et al*., 2011; Canto *et al*., 2012). In the present study, short‐term (48‐h) incubation of NMN resulted in a threefold increase in aortic NAD^+^ levels (Fig. 5A), indicating that NMN is directly taken up by the aorta and converted to NAD^+^. This is consistent with previous reports that NMN increases NAD^+^ in cultured vascular endothelial cells (Hughes‐Large *et al*., 2014) and normalizes the ratio of NAD^+^/NADH in aortic tissue (Marcu *et al*., 2015). Oral supplementation with NMN increased SIRT1 protein expression in young and tended to increase SIRT1 in old mice (Fig. 4A). We have observed an increase in SIRT1 expression previously in animals supplemented with the SIRT1 activator, SRT1720 (Gano *et al*., 2014). SIRT1 promotes autotranscriptional regulation of itself through enhanced deacetylation and activity of transcription factors, such as Forkhead transcription factor FoxO1, resulting in elevated SIRT1 protein expression (Xiong *et al*., 2011). Moreover, in the current study, supplementation with NMN restored the activity of SIRT1 in the arteries of old mice to that of young controls, as indicated by a decrease in the ratio of acetylated to total p65 subunit of the transcription factor NFκB (Fig. 4B), a well‐established target of SIRT1 (Yoshino *et al*., 2011; Gano *et al*., 2014). Although measuring the acetylation status of downstream targets of SIRT1 is an accepted method of assessing activity of the enzyme (Yoshino *et al*., 2011; Gano *et al*., 2014), protein acetylation is influenced by additional factors, such as acetyltransferases and deacetylases other than sirtuins (Peserico & Simone, 2011). Thus, it is possible that the ratio of acetylated to total p65 was due, in part, to other mechanisms. However, the current results are consistent with SIRT1 activation as the primary mechanism, as reported previously by our laboratory and others (Yoshino *et al*., 2011; Gano *et al*., 2014).

In addition to activating SIRT1, it is possible that NMN reduced oxidative stress and improved vascular function in old animals by modulating other pathways. For example, NMN may have influenced the activity of additional sirtuins, such as mitochondrial SIRT3, which is associated with decreased oxidative stress and enhanced physiological function (Someya *et al*., 2010). It is also possible that NMN enhanced metabolic flux through the tricarboxylic acid cycle and electron transport chain, thus reducing cellular reactive oxygen species (Nakamura *et al*., 2012). Moreover, NMN could have increased NADPH levels, which help maintain the glutathione and thioredoxin antioxidant systems (Nakamura *et al*., 2012). Finally, NMN may have improved other adverse influences on vascular health, such as plasma lipid profiles or blood glucose levels, thereby improving vascular function. Future investigation is warranted to study these possibilities.

## Conclusion

The results of this study provide the first evidence that oral supplementation with NMN represents a novel strategy for combating arterial aging and its pathological sequelae.

## Experimental procedures

### Animals

Young (4–8 months) C57Bl/6 male mice were purchased from Charles River, and old (26–28 months) C57Bl/6 male mice were obtained from the National Institute on Aging rodent colony. Mice were fed normal rodent chow *ad libitum* for the duration of the study. After a 2‐week acclimation period, the young and old mice were divided into two subgroups: control animals (YC, OC) continued on normal drinking water and the other animals (YNMN, ONMN) received nicotinamide mononucleotide (NMN; Sigma‐Aldrich Corp., St. Louis, MO, USA) in the drinking water (target dose of 300 mg kg^−1^ day^−1^) for 8 weeks. Body mass and water intake were monitored three times per week, and NMN concentration in the drinking water was adjusted to account for water intake. All mice were housed in an animal care facility at the University of Colorado Boulder on a 12‐hour: 12‐hour light–dark cycle. All animal procedures conformed to the *Guide to the Care and Use of Laboratory Animals* (NIH publication n. 85‐23, revised 1996) and were approved by the UCB Animal Care and Use Committee.

### Ex vivo carotid artery vasodilatory responses

Endothelium‐dependent dilation and endothelium‐independent dilation were determined *ex vivo* in isolated carotid arteries as previously described (Rippe *et al*., 2010; Sindler *et al*., 2011). Mice were anesthetized using isoflurane and euthanized by exsanguination via cardiac puncture. The carotid arteries were excised, cannulated onto glass micropipette tips, and secured with nylon (11‐0) sutures in individual myograph chambers (DMT Inc., Ann Arbor, MI, USA) containing buffered physiological saline solutions. The arteries were pressurized to 50 mmHg at 37 °C and allowed to equilibrate for 45 min before experimentation. After submaximal preconstriction with phenylephrine (2 μm), increases in luminal diameter in response to acetylcholine (ACh: 1 × 10^−9^ − 1 × 10^−4^ m; Sigma‐Aldrich Corp.) with and without co‐administration of the NO synthase inhibitor, N‐G‐nitro‐L‐arginine‐methyl ester (L‐NAME, 0.1 mm, 30‐min incubation; Sigma‐Aldrich Corp.) or the superoxide dismutase mimetic 4‐Hydroxy‐2,2,6,6‐tetramethylpiperidine‐1‐oxyl (TEMPOL, 0.1 mm, 1‐h incubation; Sigma‐Aldrich Corp.) were determined. Endothelium‐independent dilation was determined by vasodilation in response to the NO donor sodium nitroprusside (SNP: 1× 10^−10^ − 1× 10^−4^ m; Sigma‐Aldrich Corp.).

All dose–response data are presented as percent dilation as described previously (Rippe *et al*., 2010; Sindler *et al*., 2011). Preconstriction was calculated as a percent of maximal intraluminal diameter according to the following formula:

$$\text{Preconstriction}\;\left(\%\right)=\frac{D_{m}-D_{b}}{D_{m}}\times 100.$$

Because of differences in maximal carotid artery diameter between young and old animals, vasodilator responses were recorded as actual diameters expressed as a percent of maximal response according to the following formula:

$$\text{Dilation}\;\left(\%\right)=\frac{D_{s}-D_{b}}{D_{m}-D_{b}}\times 100.$$

where *D*_m_ is maximal intraluminal diameter at 50 mmHg, *D*_b_ is the steady‐state intraluminal diameter following preconstriction before the first addition of a drug, and *D*_s_ is the steady‐state intraluminal diameter recorded after the addition of a drug.

NO‐dependent dilation was determined from maximal EDD (i.e., dilation with the highest dose [1 × 10^−4^ m] ACh) in the absence or presence of l‐NAME according to the following formula:

$$\text{NO‐dependent dilation}\;\left(\%\right)=\text{Maximum}\;{\text{dilation}}_{\mathrm{ACh}}-\text{Maximal}\;{\text{dilation}}_{\mathrm{ACh}\;\;+\;\;L\;\;-\;\;\mathrm{NAME}}.$$

### *In vivo* aortic pulse wave velocity

Aortic pulse wave velocity (aPWV) was measured as described previously (Sindler *et al*., 2011; Fleenor *et al*., 2012b). Mice were anesthetized with 2% isoflurane and placed supine with legs secured to electrocardiogram (ECG) electrodes. Aortic blood flow velocity was measured with Doppler probes at the transverse aortic arch and abdominal aorta. Pre‐ejection time, the time between the R‐wave of the ECG to foot of the Doppler signal, was determined for each site. aPWV was calculated by dividing the distance between the transverse and abdominal probes by the difference in the thoracic and abdominal pre‐ejection times.

### *In vitro* elastic modulus

*In vitro* intrinsic mechanical properties of the thoracic aorta were used to calculate the elastic modulus as previously described (Fleenor *et al*., 2012b). Aortic segments (~1.5 mm), cleaned of perivascular fat and other surrounding tissue, were loaded onto a calibrated, preheated (37 °C) wire myograph chamber (DMT Inc.) containing calcium‐free phosphate‐buffered saline. The segments were prestretched for 3 min to a 1‐mm luminal diameter displacement that was returned to the nonstretched baseline, and this was repeated twice. The segments were then stretched to a baseline force of 1 mN. Luminal displacement was increased incrementally (~ 10%) every 3 min, and the force was recorded after each time period. Displacement was increased until mechanical failure of the tissue occurred, defined by an observed transient decrease in force. Stress and strain were calculated where stress was defined as: *t* = λ*L*/2*HD*. *t* = one‐dimensional stress, λ = strain, *L* = one‐dimensional load applied, *H* = wall thickness, *D* = length of vessel. Strain was defined as: λ = Δ*d*/*d*(*i*). λ = strain, Δ*d* = change in diameter, *d*(*i*) = initial diameter. The slope of the stress–strain curve was used to determine the elastic modulus as previously described (Fleenor *et al*., 2012b).

### Aortic superoxide production

Superoxide production in the thoracic aorta was measured using EPR spectroscopy, as previously described (Sindler *et al*., 2011; Fleenor *et al*., 2012b; LaRocca *et al*., 2013). One‐millimeter aortic segments, free of perivascular fat and other surrounding tissue, were incubated for 1 h at 37 °C in Krebs–Hepes buffer with the superoxide‐specific spin probe 1‐hydroxy‐3‐methoxycarbonyl‐2,2,5,5‐tetramethylpyrrolidine (CMH; 0.5 mm; Enzo Life Sciences, Inc., Farmington, NY, USA) for detection of whole‐cell superoxide production. The signal amplitude was analyzed using an MS300 X‐band EPR spectrometer (Magnettech GmbH, Berlin, Germany) with the following settings: centerfield, 3350 G; sweep, 80 G; microwave modulation, 3000 mG, and microwave attenuation, 7 dB as described previously (Sindler *et al*., 2011; Fleenor *et al*., 2012b; LaRocca *et al*., 2013). Data are presented relative to the YC group mean.

### Western blotting

Aortas were used as a surrogate large elastic artery to provide sufficient tissue for analysis of protein expression by Western blot as described previously (Sindler *et al*., 2011; Donato *et al*., 2013; LaRocca *et al*., 2013; Gano *et al*., 2014). Aortas, cleared of perivascular fat and other surrounding tissues, were frozen in liquid nitrogen before storage at −80 °C. The tissue was homogenized in ice‐cold Radioimmunoprecipitation assay (RIPA) lysis buffer containing protease and phosphatase inhibitors [Protease Inhibitor Cocktail Tablet (Roche, Indianapolis, IN, USA) and 0.01% phosphatase inhibitor cocktail (Sigma‐Aldrich Corp.)] and pulverized using a Bullet Blender. Protein was loaded on 4–12% polyacrylamide gels (12 μg per well), separated by electrophoresis, and transferred onto nitrocellulose membranes (Criterion System; Bio‐Rad Laboratories, Inc., Hercules, CA, USA) for Western blot analysis. Membranes were incubated with the following primary antibodies overnight at 4 °C: nitrotyrosine (NT 1:500; Abcam, Cambridge, MA, USA), sirtuin1 (SIRT1 1:1000; Abcam), p65 subunit of nuclear factor kappa B (NFκB; 1:500 Cell Signaling Technology Inc., Danvers, MA, USA), and acetylated p65 subunit of nuclear factor kappa B (ac‐NFκB; 1:500 Cell Signaling Technology Inc.). Proteins were visualized on a digital acquisition system (ChemiDoc‐It; UVP, Inc., Upland, CA, USA) using chemiluminescence with horseradish peroxidase‐conjugated secondary antibodies (Jackson ImmunoResearch Laboratories, Inc., West grove, PA, USA) and enhanced chemiluminescence (ECL) substrate (Pierce Biotechnology, Inc., Rockford, IL, USA). Relative intensity was quantified using ImageJ software version 1.0 (National Institutes of Health). All data were normalized to expression of alpha smooth muscle actin (α actin 1:5000; Abcam). The ratio of acetylated to total p65 was determined using two identical Western blots: one probed for acetylated p65, and the other probed for total p65. Each value was normalized to α actin in the corresponding gel. The ratio of acetylated to total p65 was determined for each animal and then normalized to the YC group mean.

### *In vitro* NMN incubation

Aortas, cleaned of perivascular fat and other surrounding tissue, were cut into equal segments and incubated in glucose‐ and nicotinamide‐free Dulbecco's modified Eagle medium (DMEM) control media (modification of Life Technologies #10566) with glucose (5 mm) and nicotinamide (1 μm) added to reflect physiologically relevant concentrations (Ramsey *et al*., 2008), with or without NMN (100 μm) for 48 h, and then either immediately frozen in liquid nitrogen for assessment of aortic NAD^+^ concentration, or embedded and frozen in optimal cutting temperature compound (OCT; Fisher Scientific Inc., Waltham, MA, USA) for assessment of MnSOD expression using immunohistochemistry as described below.

### NAD^+^ measurement

Aortic NAD^+^ levels were determined in young (12 month) mice using an HPLC system (Shimadzu, Kyoto, Japan) with a Supelco LC‐18‐T column (15 cm × 4.6 cm; Sigma‐Aldrich Corp.) as described previously (Yoshino *et al*., 2011).

### Immunohistochemistry

Immunohistochemistry was used to determine aortic expression of collagen type I, elastin, and MnSOD as previously described (Fleenor *et al*., 2010, 2012b). Thoracic aorta segments, cleared of perivascular fat and other surrounding tissue, were frozen in OCT compound in liquid nitrogen‐cooled isopentane. Seven‐micrometer aortic segments were fixed in acetone for 10 min and washed in Tris buffer. All slides were stained with the Dako EnVision + System‐HRP‐DAB‐kit according to the manufacturer's protocol using the following primary antibodies: collagen type 1 (Col I; 1:100, Millipore Corp., Temecula, CA, USA), alpha elastin (α elastin; 1:50, Abcam), and MnSOD (1:500, StressGen Biotechnologies). Primary antibodies were incubated for 1 h at 4 °C. The labeled polymer was applied for 30 min, and staining was visualized after a 4‐min exposure to diaminobenzidine (DAB). Slides were then dehydrated and coverslipped. Digital photomicrographs were obtained using a Nikon Eclipse TS100 photomicroscope, and quantification was performed with ImageJ software version 1.0. Slides from multiple batches were normalized to the same representative YC/vehicle animal and normalized to the YC/vehicle mean of each staining day.

### Statistical analyses

Data are presented as mean ± SEM in text, figures, and tables. All analyses were performed with SPSS. Student's *t*‐test was used to analyze aortic NAD^+^ and MnSOD. A one‐way ANOVA was used to analyze morphological characteristics, maximum EDD, NO‐mediated EDD, EPR spectroscopy, aPWV, elastic modulus, Western blots, and immunohistochemistry. Within‐group differences in the maximal EDD dose–response to acetylcholine in the absence vs. presence of TEMPOL was determined using two‐factor (group × treatment) repeated‐measures ANOVA. When a significant main effect was observed, Tukey *post hoc* tests were used to determine specific pairwise differences. Significance was set at *P* < 0.05.

## Author contributions

L.B.G., S.I., and D.R.S. contributed to the conception of the study, and all authors designed experiments. N.E.de P. and L.C.J. collected the majority of data. N.E.de P., A.L.S., L.C.J., C.R.M., and K.F.M. contributed to data analysis. All authors contributed to data interpretation. N.E.de P., C.R.M., and D.R.S. drafted the manuscript, and all authors critically revised and provided final approval of the submitted version.

## Funding

This work is supported by the National Institutes of Health (AG013038, AG000279 to D.R.S., and AG024150, AG037457 to S.I.) and was conducted while C.R.M. was a Glenn/AFAR Postdoctoral Fellow.

## Conflict of interest

S.I. is a co‐founder of Metro Midwest Biotech. N.E.de P., L.B.G., A.L.S. and D.R.S. filed the PCT Application number PCT/US15/22267, METHODS FOR TREATMENT OF VASCULAR ENDOTHELIAL DYSFUNCTION USING NICOTINAMIDE MONONUCLEOTIDE on March 24, 2015. All other authors declare that they have no conflicts of interest.
